# *In Situ* and *Ex Situ* Silver Nanoparticle Modification of Lyocell Fibers: Insights into Nanoparticle Size Control and Physicochemical Properties

**DOI:** 10.3390/ma19132736

**Published:** 2026-06-26

**Authors:** Emilia Śmiechowicz, Michalina Stefaniak

**Affiliations:** Department of Mechanical Engineering, Informatics and Chemistry of Polymer Materials, Faculty of Textiles and Design, Lodz University of Technology, 116 Żeromskiego Street, 90-924 Lodz, Poland; 241270@edu.p.lodz.pl

**Keywords:** Lyocell-type cellulose fibers, N-methylmorpholine N-oxide, *in situ* silver nanoparticles generation, *ex situ* silver nanoparticles synthesis, glucose reduction, nanoparticle size control

## Abstract

Controlling the parameters of nanoparticles within a polymer matrix is a key challenge in advancing modern materials science, particularly in developing functional cellulose-based materials. This study focused on a comprehensive comparison of *in situ* and *ex situ* methods for synthesizing silver nanoparticles (AgNPs) to determine the optimal approach for achieving precise control over nanoparticle size and distribution within Lyocell-type fibers. Cellulose fibers were produced via the NMMO (N-methylmorpholine N-oxide) method, using glucose as an eco-friendly reducing agent for silver nitrate in both approaches. The *in situ* method involved generating AgNPs directly during spinning dope preparation, whereas the *ex situ* approach utilized chemical reduction under various conditions (12 h and 24 h, 70 °C) prior to incorporating the pre-synthesized nanoparticles into the fibers. UV-Vis, DLS, and TEM measurements were employed to characterize the nanoparticles, while the resulting fibers were evaluated for their degree of cellulose polymerization, as well as their mechanical and hygroscopic properties. The comparative analysis revealed that the *ex situ* synthesis (24 h, 70 °C) is the optimal method, enabling superior control over the nanoparticle parameters and successfully introducing a high percentage of small-diameter AgNPs (2–8 nm) into the fiber matrix without degrading the fundamental properties of the cellulose.

## 1. Introduction

In recent years, the demand for regenerated cellulose fibers has grown rapidly, not only due to their relative environmental friendliness compared to cotton and viscose, but also due to the unique properties of the fibers obtained from cellulose. To manufacture these fibers, high-purity cellulose pulp is isolated from biomass via chemical pulping (e.g., Kraft or sulfite processes). In the Lyocell method, this pulp is directly dissolved in N-methylmorpholine N-oxide (NMMO) and extruded into an aqueous bath to precipitate cellulose filaments in a closed-loop system, which achieves over 99% solvent recovery [1]. Due to their desirable morphological properties and functional versatility during the production process, Lyocell fibers have found applications in many industrial areas, including as a raw material for a wide range of textile applications, e.g., fire resistant, luminescent, biodegradable, thermoregulating with phase-change materials (PCMs), and as temperature sensors [2,3,4,5,6,7,8,9,10,11,12,13,14]. Lyocell fibers and materials have well-documented biodegradable properties [9]. The research conducted on this topic in recent years has provided further valuable information. Specifically, Lyocell fibers are characterized by a rapid surface-level degradation process, but slower-than-expected microbial penetration [10]. Despite this, a group of scientists studying nonwoven fabrics containing Lyocell (LY) fibers, led by the Technological Center of the Textile and Clothing Industry in Portugal [11], demonstrated that LY nonwovens completely degraded after 55 days of exposure to soil. The authors also demonstrated that the biodegradation process of nonwoven fabrics had no toxic effects on the soil, which shows the great potential of using Lyocell fibers in the production of nonwoven fabrics for ecological and biodegradable applications. Due to their natural biocompatible properties, Lyocell fibers are often used as a base fiber for modifications, such as physical treatments (introducing active compounds into the spinning solution), chemical treatments (through the reaction of functional groups of compounds with the fiber material, conducting grafting or cross-linking processes), and post-treatment (applying antibacterial compounds and depositing them on the fiber surface through physical coating or impregnation processes) [15]. The extensive interest in silver nanoparticles is primarily driven by their capacity to enhance the physicochemical and structural performance of textile materials [16,17].

In the case of textile materials, their modification with silver nanoparticles can be carried out by using *in situ* methods (generation of nanoparticles in contact with the fiber matrix) or *ex situ* (modification of the textile material with ready-made nanoparticles obtained by separate nanoparticle synthesis). In both of these methods, controlling the particle size and assembly during the direct modification of fibers remain key challenges. Recently, this was demonstrated by systematic studies on the reactive *in situ* synthesis of self-assembled AgNPs on natural yarns; by precisely optimizing these parameters, the authors successfully controlled the size range of AgNPs on cotton yarn from 10 to 50 nm [18]. Conversely, the *ex situ* approach remains highly relevant for green chemistry processes, during which pre-synthesized biogenic silver nanoparticles are integrated into structured fiber matrices. In this context, recent research has focused on the development of polymer nanocomposites based on biologically synthesized silver nanoparticles (AgNPs) and polyvinyl alcohol (PVA). In one study, silver nanoparticles were prepared using an aqueous extract of dried lavender leaves (*Lavandula angustifolia*), with more than 70% having a diameter less than 20 nm [19]. In another study, a method for the post-photosynthesis of silver nanoparticles through the chemical reduction of silver nitrate with stannous chloride under daylight irradiation using CTAB as a stabilizer was developed [20]. This research demonstrated a new method for the *ex situ* and *in situ* post-photosynthesis of silver (Ag) nanoparticles on fabric polyamide surfaces at room temperature. This method can be applied to various substrates for the *in situ* photosynthesis of silver nanoparticles whenever required.

Previously, nanocomposite Lyocell cellulose fibers modified with silver nanoparticles and nanosilica encapsulated in a cellulose matrix were developed [4]. The silver nanoparticles were generated by chemically reducing silver nitrate (AgNO_3_) in a 50% aqueous solution of NMMO (*ex situ* method), which was also used as the direct solvent for the cellulose pulp used to produce the Lyocell fibers. That study focused on analyzing both the fundamental physical parameters of the nanoparticles and the structural properties of the resulting fibers [4]. A group of scientists [5] also obtained antibacterial Lyocell fibers with nanosilica and immobilized silver nanoparticles. Knitted structures were present in the selected fibers. The structural and physical parameters of the resulting knitted fabrics were examined, and indicated that the nanomodifier used in the fibers did not significantly affect the knitted fabrics’ physical properties. Furthermore, the results of studies on the water vapor permeability, cut resistance, and pH in relation to the functional and protective properties of interest to the authors, as well as user comfort, showed that the resulting knitted fabrics could be used in the production of bioactive protective gloves [21].

Rigo et al. [22] conducted research on a flexible polyethylene knit fabric coated with silver nanoparticles. Their study demonstrated that such structural configurations are highly relevant for engineering functional surfaces. Textiles modified with silver nanoparticles are also used in protective applications and advanced materials. Ali et al. [23] successfully incorporated a mixture of silver nanoparticles and titanium dioxide onto Egyptian cotton fabrics using a padding and drying method. These textiles can be successfully used as protective clothing.

This study investigates the introduction of silver nanoparticles (AgNPs) into a Lyocell fiber matrix using two distinct approaches: the *in situ* method (direct generation of nanoparticles during the spinning dope preparation) and the *ex situ* method (pre-synthesis of nanoparticles using glucose as an eco-friendly reducing agent under varying conditions (12 h and 24 h at 70 °C), followed by their introduction into the cellulose/NMMO system). While AgNPs are selected as the model bioactive agent due to their well-established broad-spectrum antimicrobial efficacy, this study specifically addresses the structural challenges of their integration into a polymer matrix.

The fundamental objective of this study is to perform a comprehensive, comparative evaluation of these two synthesis methodologies. By systematically investigating the impact of *in situ* versus *ex situ* approaches on nanoparticle parameters (such as diameter, shape, stability, and distribution) and the subsequent properties of the modified Lyocell fibers (including degree of polymerization, mechanical performance, and hygroscopicity), this work aims to identify the optimal processing route for achieving precise structural control.

The broader significance of this research lies in its practical contribution to advanced materials science and engineering, specifically within the functionalization of cellulose-based matrices. Establishing a controllable and optimized method for introducing ultra-small AgNPs (2–8 nm size range) without degrading the fundamental structural properties of cellulose offers substantial application potential. Such bioactive modification of cellulose fibers represents a highly promising strategy for biomedical applications [24]. Ultimately, these findings will provide a scalable and sustainable technological framework for the precise development of structurally optimized matrices, offering a valuable starting point for the future design of high-performance, biologically active biomaterials, such as advanced wound dressings capable of effectively combating a wide range of pathogens.

## 2. Materials and Methods

### 2.1. Materials

In this study, the following materials and chemical reagents were used to produce Lyocell cellulose fibers modified with silver nanoparticles:

- Cellulose pulp (Rayonier^®^, Rayonier Inc., Wildlight, FL, USA) was used, which was characterized by an α-cellulose content of 98.36%, polymerization degree of DP = 1.236, and moisture content of 5.92%.
- A 50% aqueous solution of N-methylmorpholine-N-oxide (NMMO) manufactured by the HUNTSMAN Corporation Belgium NV^®^ (Tienen, Belgium) was used as the direct solvent.
- Tenox PG (propyl ester of gallic acid) (Sigma^®^, St. Louis, MO, USA) was used as an antioxidant.
- Silver nitrate (AgNO_3_) manufactured by CHEMPUR^®^ (Piekary Śląskie, Poland), was used as the substrate for the synthesis of AgNPs.
- Glucose (C_6_H_12_O_6_) from Sigma Aldrich^®^ (St. Louis, MO, USA) was used as an eco-friendly reducing agent in the production of silver nanoparticles.

### 2.2. Methods

#### 2.2.1. Preparation of Chemical Reagents for Obtaining AgNPs

A green (pro-ecological) method for obtaining silver nanoparticles was used. An aqueous solution of silver nitrate and glucose, each at a concentration of 0.01 mol/dm^3^, was prepared. Appropriate amounts of the dissolved components (silver nitrate and glucose) were measured, to which distilled water was then added to achieve the desired concentration. The measured mass of the components resulted from the molar ratio of silver to glucose (2:1) involved in the reaction. The aqueous solution of silver nitrate was stored in a darkroom at a reduced temperature to prevent photochemical decomposition. This metal precursor was used to produce the AgNPs, which were added in an amount that allowed for an in-fiber concentration of 0.05% (500 ppm). This concentration allowed for the production of cellulose fibers modified with silver nanoparticles with antibacterial properties [25].

#### 2.2.2. *Ex Situ* and *In Situ* Production of Silver Nanoparticles

One of the methods used in this study was an *ex situ* technique involving the synthesis of AgNPs in a separate system (NMMO–AgNO_3_–glucose). The AgNPs were prepared through a chemical reaction by reducing silver ions, with glucose as the reducing agent. The reaction proceeded according to the following scheme [26,27]:

(1) $$2\;{\mathrm{Ag}}^{+}+C_{6}H_{12}O_{6}+H_{2}O\;\to \;2\;{\mathrm{Ag}}^{0}+C_{6}H_{12}O_{7}+2\;H^{+}$$

The previously prepared glucose solution was introduced into a previously measured NMMO solution, to which silver nitrate was then added dropwise. In this prepared system, the AgNPs were synthesized at 70 °C in a darkroom, with reaction times of 12 and 24 h, and the synthesized AgNPs were then incorporated into the components of spinning dope. In the second method—*in situ*—the nanoparticles were obtained through direct contact with cellulose and NMMO during the preparation of a spinning dope for fiber production. Previously prepared glucose and silver nitrate solutions, with the molar ratio maintained, were then added directly into a laboratory Ikavisk blender along with the other components of the spinning dope at the beginning of the cellulose dissolution process. In this case, both the cellulose and glucose were reducing agents for the AgNPs.

The silver nanoparticles are designated as

AgNPs—silver nanoparticles generated *in situ* and synthesized *ex situ*.

The designations for the NMMO solutions with silver nanoparticles:

S 0—Reference solution, 50% NMMO without modifier;

S-12/70—NMMO solution with AgNPs, synthesized *ex situ* for 12 h at 70 °C;

S-24/70—NMMO solution with AgNPs, synthesized *ex situ* for 24 h at 70 °C.

#### 2.2.3. Fiber-Forming Process

The fibers were spun at a speed of 55 rpm using the dry–wet method on a laboratory spinning machine, which was described in previous work [28].

The designations for the cellulose fibers:

F 0—Standard cellulose fiber unmodified with AgNPs;

F Ag/1—Cellulose fiber with AgNPs generated directly in a laboratory mixer using glucose;

F Ag 12/70/G—Cellulose fiber with AgNPs synthesized *ex situ* in the NMMO

–AgNO_3_–glucose system for 12 h and at 70 °C;

F Ag 24/70/G—Cellulose fiber with AgNPs synthesized *ex situ* in the NMMO

–AgNO_3_–glucose system for 24 h and at 70 °C.

### 2.3. Methods for Testing the Properties of AgNPs and Fibers Modified with AgNPs

#### 2.3.1. UV/Vis Spectroscopy

To monitor the formation and progress of AgNPs synthesis, a Jasco V-570 UV-Vis spectrophotometer (JASCO INTERNATIONAL Co., Tokyo, Japan) was utilized within a scanning range of 250–700 nm, and the data were collected using the manufacturer’s dedicated software. The obtained spectral data were processed and analyzed using Microsoft Excel software (Microsoft 365 for business, Microsoft Corporation, Redmond, WA, USA). Prior to measurement, the nanoparticle dispersions in the NMMO media underwent a 1:1 dilution with distilled water. An aqueous solution of NMMO, prepared at the identical dilution ratio, served as the blank reference.

#### 2.3.2. Dynamic Light Scattering (DLS)

The evaluation of silver nanoparticle dimensions and volume-based size distributions were executed via dynamic light scattering (DLS). These measurements were performed using a PSS Nicomp 380 particle sizer (PSS NICOMP, Santa Barbara, CA, USA). The data processing and size analysis were completed using the CW 388 application within the PSS Nicomp software framework (v. 1.55).

#### 2.3.3. Transmission Electron Microscopy (TEM)

Morphological assessment of the synthesized nanoparticles was conducted at the Institute of Metallurgy and Materials Science of the Polish Academy of Sciences (IMMS PAS) in Kraków using a TECNAI SuperTWIN FEG (200 kV) transmission electron microscope (FEI Co., FEI Electron Optics B.V., Eindhoven, Noord-Brabant, The Netherlands). Structural features of the modified fibers were investigated under both bright- and dark-field modes using a dedicated TEM microscope setup (P/19/IB-05, issue 3, 25 July 2003). For detailed analysis of AgNPs shape and size profiles, high-resolution (HR) along with standard bright-field (BF) techniques (procedure P/19/6 IB-05) were deployed. Quantitative evaluation of these geometric properties was carried out using NIS-ELEMENTS software (BR 4.13.05 64-bit), Nikon, Tokyo, Japan.

#### 2.3.4. Average Degree of Polymerization ($\bar{DP}$) of Cellulose

The viscometric technique involving an Ubbelohde dilution viscometer (SI Analytics, Mainz, Germany) was employed to establish the average degree of polymerization ($\bar{DP})$ of cellulose within the fiber matrices. All procedures strictly adhered to the protocol outlined in the ISO 5351:2010 standard [29].

#### 2.3.5. Hygroscopic Parameters of Fibers

Moisture absorption of the obtained fibers at 65% RH and 20 °C was determined in accordance with the standard textile testing atmosphere defined in ISO 139:2005 [30]. The water retention value (WRV) was measured in accordance with the international standard ISO 23714:2014 [31]. 

#### 2.3.6. The Mechanical Parameters of the Obtained Fibers

The fiber linear density evaluations followed the guidelines of the ISO 1973:2021 (E) standard [32]. Tensile testing, specifically the determination of conditioned tenacity and elongation at break, was executed in compliance with PN-EN ISO 5079:2020 [33]. The mechanical characterization was conducted on a ZWICK/Z 2.5/TN1S tensile testing platform (ZwickRoell, Ulm, Germany) controlled by TestXpert v. 7.1 software.

## 3. Results and Discussion

### 3.1. Organoleptic Characteristics of Fibers

One of the most organoleptically differentiating features of the obtained cellulose fibers is their color. Standard fibers, designated F 0 (unmodified), are pure white. However, fibers modified with AgNPs vary in color depending on the modification method. F Ag/1 fibers, generated directly in a laboratory kneader in the presence of cellulose and glucose, feature a gray hue. The fibers with AgNPs generated in the NMMO–AgNO_3_–glucose system at 70 °C have a warm hue. The variation in color intensity is influenced by the synthesis time. The fibers with nanoparticles synthesized for a shorter time (12 h), designated as F Ag 12/70/G, have a warmer, slightly orange shade, while the fibers synthesized for a longer time (24 h), designated as F Ag 24/70/G, exhibit a significantly lighter, golden color. Previous studies [34] have shown that the diameter and shape of silver nanoparticles have a significant influence on the color of the obtained cellulose fibers. This is mainly related to the intensity of plasmon phenomena, the distribution of nanoparticles in the polymer matrix, and the interactions between the nanoparticles and the surrounding polymer. A comparison of the colors of all the obtained fibers is presented in Table 1 below.

### 3.2. Methods for Testing the Properties of AgNPs and Fibers Modified with AgNPs

#### 3.2.1. Analysis of the Progression of *Ex Situ* AgNPs Synthesis Using UV/Vis

**Figure 1:**
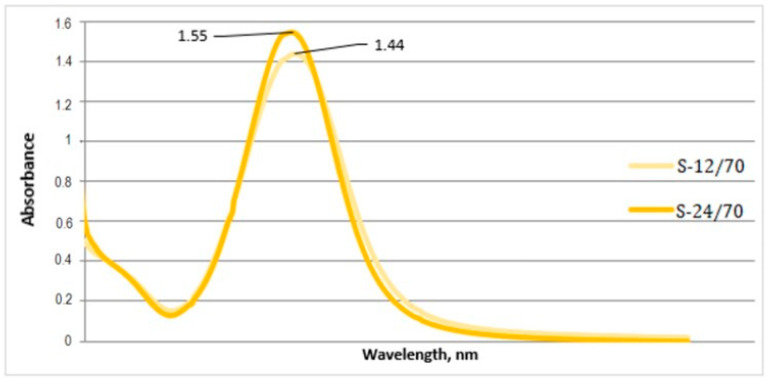
Comparison of the relationship between the absorbance and the wavelength of AgNPs synthesized *ex situ* in NMMO with variable times. The abbreviations correspond to the following: S—NMMO solution. Numbers 12/70 represent synthesis time (h) and temperature (°C), respectively.

The degree of conversion of the nanoparticle precursor in the NMMO–AgNO_3_–glucose system, depending on the *ex situ* synthesis process duration (12 or 24 h), was examined using UV-Vis spectrophotometry. A 50% aqueous solution of NMMO served as the reference substance. Photographs of the obtained solutions are presented below (Table 2), along with the absorbance measurement results for the analyzed systems (Figure 1). Referring to the organoleptic assessment of the nanoparticles obtained in NMMO, the photographs presented in Table 2 show that the solutions with AgNPs have a significantly darker color compared to the reference solution (50% NMMO without nanoparticles). However, in the case of the solution with nanoparticles synthesized over a longer period (24 h), a deeper color intensity and a darker color can be observed compared to the solution synthesized over a shorter period (12 h).

Based on the obtained spectrograms (Figure 1), it can be seen that the absorbance maximum falls in a range around 400–450 nm, which confirms the successful synthesis process and the presence of AgNPs in NMMO. A greater degree of AgNO_3_ conversion in NMMO is observed for the longer synthesis time (24 h), as the absorbance of 1.55 for sample S-24/70 is noticeably higher compared to the absorbance of 1.44 for sample S-12/70. The increase in absorbance indicates a higher proportion of AgNPs in the solution for which a longer synthesis time was used (S-24/70), which is also confirmed by the darker color of the solution (Table 2).

#### 3.2.2. Multi-Modal Size Distribution of AgNPs: Volume- and Number-Weighted Analysis Using DLS

In order to investigate the diameters of nanoparticles synthesized *ex situ* in NMMO–AgNO_3_–glucose systems for 12 h and 24 h, dynamic light scattering (DLS) measurements were performed, which are presented below in Table 3.

Analyzing the measurement results presented in Table 3, it can be observed that for the reference solution S 0, only one fraction assigned to NMMO is visible. In the S-12/70 solution, after 12 h of synthesis, three fractions of nanoparticles were isolated—in fraction 1, particles with a diameter of 11.6 nm were observed, while in fractions 2 and 3, agglomerates or aggregates of nanoparticles with diameters of 119.7 nm and 912 nm were observed, respectively. The largest share of the total percentage volume of nanoparticles (95.3%) was held by the particles with the smallest diameter of approximately 11.6 nm. In the S-24/70 solution, after 24 h of synthesis, the nanoparticles with the smallest diameter of approximately 12.6 nm also constituted the largest volume (83.1%) in the population, assigned to fraction 1. It can also be noticed that in the S-24/70 solution, only one fraction of agglomerates and aggregates with a diameter of approximately 90 nm was present. A longer nanoparticle synthesis process not only results in the generation of particles with greater dimensional uniformity (only two fractions), but also reduces the volume fraction of single particles (from 95.3% to 83.1%).

Nanoparticle size measurements using DLS were also performed on dissolved fibers modified with nanoparticles synthesized *ex situ* in the solutions analyzed above and with nanoparticles generated *in situ* directly with the spinning dope components. DLS measurements were also performed on the unmodified F 0 reference fiber. The measurements obtained for the above fibers are summarized in Table 4.

The results for the reference fiber (F 0) presented in Table 4 show that, as expected, there were no AgNP fractions. The only detected fraction was one with a particle diameter of 3.8 nm, indicating the presence of undissolved cellulose clump [35].

Two nanoparticle fractions were detected in the fiber with nanoparticles synthesized using the *in situ* method (F Ag/1): the fraction 1 particles had a diameter of 22.1 nm and the fraction 2 particles had a diameter of 1763.5 nm, indicating the presence of nanoparticle aggregates or agglomerates within the fiber structure. The vast majority of the nanoparticles generated in the fiber were single particles, occupying as much as 98.9% of the system’s volume.

In both cases of *ex situ* synthesis of nanoparticles in the NMMO–AgNO_3_–glucose system, the smallest nanoparticles constituted the vast majority of AgNPs present in the fibers (nearly 100% in terms of number weighting). The study also reveals a high volume fraction of nanoparticles of this size in the F Ag 12/70/G and F Ag 24/70/G samples—98.7% and 99.8%, respectively. Agglomerates of nanoparticles are present in both samples. In the F Ag 12/70/G sample, two fractions are visible—a second fraction of particles with a diameter of 318.7 nm, the share of which is negligible (less than 0.1%), and a third fraction of particles with a diameter of 3054.3 nm, which constitutes 1.3% of the detected particles. In the F Ag 24/70/G sample, the study reveals one fraction of nanoparticle agglomerates with a diameter of 1595.6 nm, with a small share (0.2%).

Comparing the results obtained for the AgNPs in fibers synthesized using different methods, it can be concluded that in the case of *ex situ* synthesis, a longer process time (24 h) is responsible for increased homogeneity of detected particles, as evidenced by the lower number of detected fractions. Furthermore, a longer synthesis time of nanoparticles results in a slightly higher proportion of individual nanoparticles in the fiber volume—99.8% compared to 98.7% for particles synthesized using shorter times. A longer synthesis time also has a beneficial effect on reducing the size of nanoparticle agglomerates, reducing their diameter by approximately half.

The *ex situ* synthesis of particles in a separate system also promoted the formation of smaller nanoparticles (ca. 12 nm) than the *in situ* generation of AgNPs (ca. 22 nm), which produced nanoparticles almost twice as large in the fiber matrix. Using cellulose as one of the nanoparticle reducers in the *in situ* method promoted the formation of particles above 20 nm, as demonstrated in research conducted previously by Smiechowicz et al. [28]. Despite the positive effect of *in situ* synthesis on particle homogeneity (only two fractions), the agglomerates or aggregates formed were significant in size (ca. 1800 nm) and had a larger share of the total population volume (1.1%) compared to the F Ag 24/70/G fiber (0.2%). Comparing the data in Table 3 and Table 4, it can be concluded that the methods used for AgNPs production significantly affected the morphology of silver nanoparticles present in the fibers. Despite the noticeable increase in agglomerates in the fibers, there was a clear dominance of single nanoparticles (their substantial shares). This was particularly visible in the F Ag 24/70/G sample modified with nanoparticles obtained *ex situ* after 24 h of synthesis, where the share of the monodisperse fraction in the fiber increased by 16 percentage points compared to the S-24/70 starting solution.

#### 3.2.3. Microstructural Analysis of AgNPs in Fibers Using TEM

**Figure 2:**
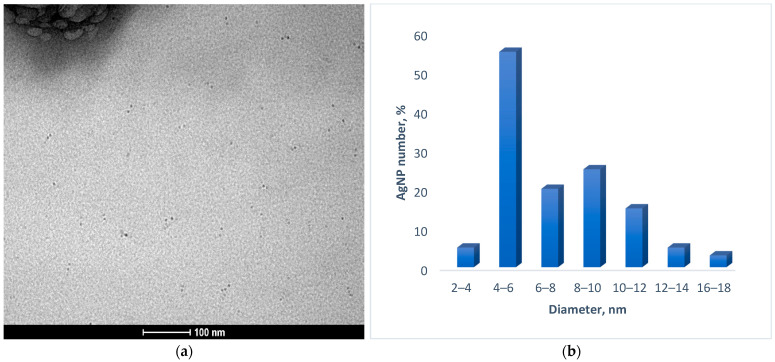
(**a**) TEM image of AgNPs enclosed in fiber F Ag 12/70/G and (**b**) AgNPs size distributions. The abbreviations correspond to the following: F—cellulose fiber; Ag—silver nanoparticles; G—glucose. Numbers 12/70 represent synthesis time (h) and temperature (°C).

**Figure 3:**
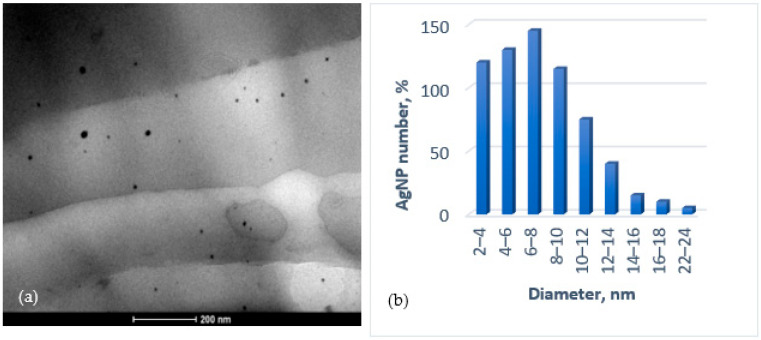
(**a**) TEM image of AgNPs enclosed in fiber F Ag 24/70/G and (**b**) AgNPs size distributions. The abbreviations correspond to the following: F—cellulose fiber; Ag—silver nanoparticles; G—glucose. Numbers 24/70 represent synthesis time (h) and temperature (°C).

The polymer matrix structures of the selected fibers, modified with *ex situ* AgNPs, were analyzed using a transmission electron microscope (TEM). This method allowed for imaging of the nanoparticles and a precise assessment of their morphology, size, and degree of dispersion within the fibers, which significantly complemented the results obtained by DLS. Below are selected bright-field (BF) TEM images of the selected fibers, which were used to generate graphs of the nanoparticle size distribution by particle population (Figure 2 and Figure 3). To observe the nanoparticle shape, Figure 3a also presents a high-resolution (HR) image of silver nanoparticles in the fibers.

**Figure 4:**
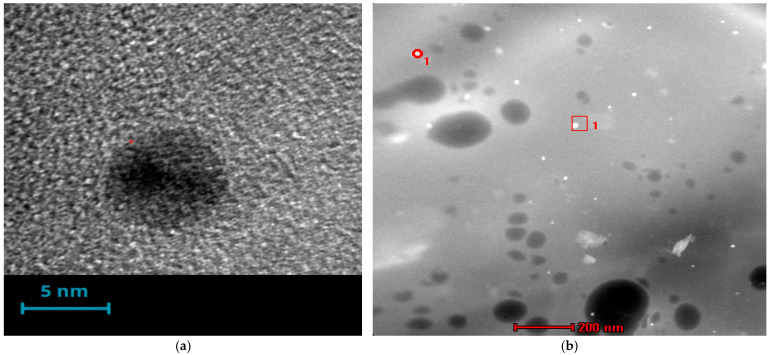
(**a**) HR image of a nanoparticle in an F Ag 24/70/G fiber. (**b**) Dark-field TEM image of a fiber The red squares/dotes indicate the regions where AgNPs were identified. The abbreviations correspond to the following: F—cellulose fiber; Ag—silver nanoparticles; G—glucose. Numbers 12/70 represent synthesis time (h) and temperature (°C).

Analyzing the nanoparticle size distributions in the fibers, it can be seen that in the F Ag 12/70/G fiber there is a clear predominance of nanoparticles with sizes ranging from 4 nm to 6 nm, while in the F Ag 24/70/G fiber, most nanoparticles are between 6 nm and 8 nm. It is worth noting that in the F Ag 24/70/G fiber, a significant predominance of nanoparticles with small sizes ranging from 2 nm to 8 nm is observable, with extremely small particles with diameters of 1–2 nm also visible. The BF and HR images show an optimal degree of homogeneity of distribution, maintaining a relatively uniform spacing between spherical nanoparticles (Figure 4), without noticeable aggregates or agglomerates of particles.

**Figure 5:**
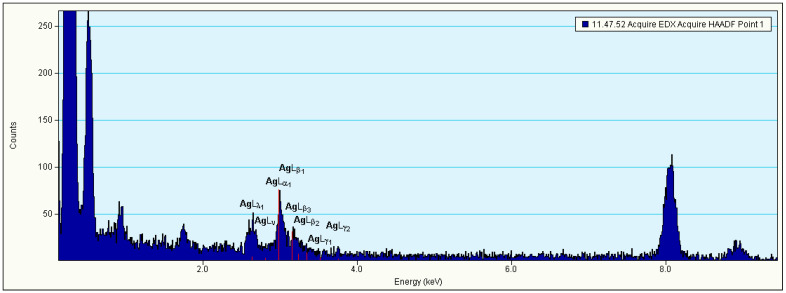
TEM-EDS spectrum of F Ag 24/70/G fiber. The abbreviations correspond to the following: F—cellulose fiber; Ag—silver nanoparticles; G—glucose. Numbers (e.g., 24/70) represent synthesis time (h) and temperature (°C).

To confirm the presence of AgNPs in the fibers, an X-ray diffraction (EDS) microanalysis was performed using the TEM technique. A chemical composition analysis was performed for the red-marked areas of the selected F Ag 24/70/G fibers (Figure 4b) and is presented in Figure 5 below.

The TEM-EDS examination confirmed the presence of AgNPs in the F Ag 12/70/G cellulose fiber matrix, as evidenced by the presence of clear silver peaks visible in the TEM-EDS spectrum above (Figure 5).

The TEM and TEM-EDS measurements confirmed the presence of silver nanoparticles in the fiber matrices, which are consistent with the DLS results. Furthermore, the TEM method supplemented the results by presenting an actual image of the nanoparticles in the fiber matrix (size, shape, and distribution within the fiber matrix). The TEM analysis revealed the presence of nanoparticles in the 1–10 nm range, undetected by DLS. This indicates the need for the complementary use of both research techniques to obtain a complete profile of particle size classes, particularly in the case of polydisperse nanoparticle systems in fibers.

#### 3.2.4. Average Degree of Polymerization ($\bar{DP}$) of Cellulose Analysis in Fibers

The aim of the $\bar{DP}$ measurement was to verify the effect of nanoparticles generation conditions and the presence of AgNPs in the fibers on the changes in the average degree of cellulose polymerization in the prepared samples. Based on this, the effect of the modification on the potential degradation of cellulose in the fiber could be investigated. The measurement results are presented in Table 5.

It can be seen that the $\bar{DP}$ results obtained for the modified fibers differ only slightly from the value obtained for the unmodified fiber. The $\bar{DP}$ variations of approximately 15 were within the measurement error, which may have also been influenced by the slight unevenness in the cellulose pulp used for fiber production. It can be concluded that the methods used to obtain the nanoparticles and modify the fibers did not negatively affect the stability of the polymer chain in the fibers. This indicates no degradation of the cellulose in the fibers.

#### 3.2.5. Analysis of Hygroscopic Parameters of Fibers

**Figure 6:**
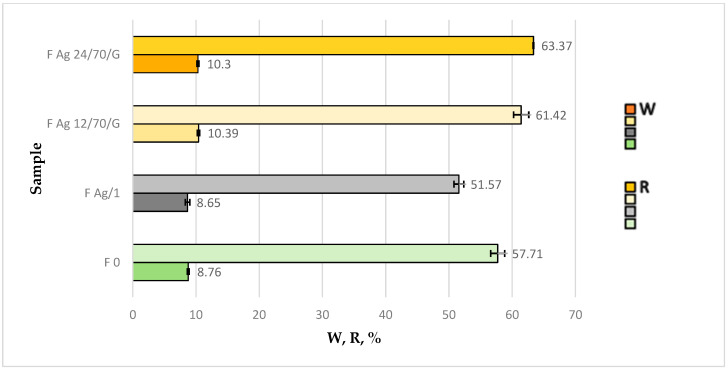
Moisture absorption (W) and water retention (R) of AgNP-modified and unmodified fibers. The abbreviations correspond to the following: F—cellulose fiber; Ag—silver nanoparticles; G—glucose. Numbers (e.g., 24/70) represent synthesis time (h) and temperature (°C), respectively. Error bars represent standard deviation (SD).

The moisture absorption (W) and water retention (R) capacities of the fibers were assessed to analyze the impact of silver nanoparticle generation methods and cellulose matrix modification on fiber hygroscopicity. Fiber hygroscopicity studies were also conducted to investigate the potential suitability of the fibers for the production of wound dressings. The test results for the modified and unmodified fibers are presented in a summary graph (Figure 6).

It can be concluded that modifying fibers with silver nanoparticles by directly generating them in a laboratory blender slightly reduced the values of the tested hygroscopic parameters; i.e., the moisture absorption was reduced by 0.11 percentage points and the water retention was reduced by 6.14 percentage points compared to the unmodified fibers.

The fibers with silver nanoparticles synthesized *ex situ* in NMMO–AgNO_3_–glucose systems achieved better properties; specifically, the fibers modified with AgNPs synthesized for 12 h exhibited 1.63 percentage points higher moisture absorption and 3.71 percentage points higher water retention than the standard fibers, while the fibers modified with AgNPs synthesized for 24 h exhibited 1.54 percentage points higher moisture absorption and 5.66 percentage points higher water retention than the standard fibers. The *ex situ* method of obtaining AgNPs and modifying the fibers with them resulted in fibers with better hygroscopic properties than the *in situ* method. The F Ag 24/70/G fiber demonstrated the highest water retention, making it suitable for potential medical applications. Although these enhanced hygroscopic properties are crucial for wound care, a comprehensive biological and antimicrobial evaluation was beyond the scope of this study, which focused strictly on material characterization.

#### 3.2.6. Analysis of Mechanical Parameters of Fibers

The aim of studying the basic mechanical parameters of fibers was to estimate the influence of the applied methods of generating nanoparticles on the conditioned tenacity and elongation at break of the fibers. The mechanical parameters of modified and unmodified fibers are presented in Table 6.

In the case of modification of the fiber matrix with the nanoparticles generated *in situ*, the F Ag/1 fibers were characterized by the lowest decrease in conditioned tenacity (less than 4 cN/tex) and the lowest elongation at break (about 1.5%) in relation to the standard fibers. In contrast, the fibers with the nanoparticles synthesized *ex situ* exhibited lower conditioned tenacity values (5-6 cN/tex) and an elongation at break lower than 1–1.2%. The differences between the mechanical parameter values for the modified fibers and the standard fibers (strength: 4-6 cN/tex; elongation: 1–1.5%) were not significant, especially in relation to the potential application of fibers with nanoparticles as wound dressing materials.

## 4. Conclusions

The study successfully demonstrates the fabrication of Lyocell-type cellulose fibers functionalized with silver nanoparticles (AgNPs) using two eco-friendly, glucose-mediated methods. The following main conclusions can be drawn:

- Both *ex situ* and *in situ* functionalization methods allow for efficient incorporation of AgNPs without inducing degradation of the cellulose polymer chains or significantly altering the essential mechanical properties of the fibers.
- The *ex situ* approach facilitates the formation of smaller, highly dispersed spherical nanoparticles (2–12 nm) with low agglomeration tendencies, which effectively enhance the hygroscopic properties (moisture absorption and water retention capacity) of the modified fibers.
- Fibers modified via the *ex situ* method exhibit optimal structural and sorption characteristics, making them promising candidates for advanced medical applications, particularly in the production of functional wound dressing materials.

Future research is planned to include preliminary biological assays to evaluate the developed materials for potential wound care applications.

## Figures and Tables

**Table 1:** Lyocell-type cellulose fibers modified with silver nanoparticles and standard, unmodified fibers. The abbreviations correspond to the following: F—cellulose fiber; Ag—silver nanoparticles; G—glucose. Numbers 12/70 represent synthesis time (h) and temperature (°C), respectively.

Samples	F 0	F Ag/1	F Ag 12/70/G	F Ag 24/70/G
Color comparison of fibers	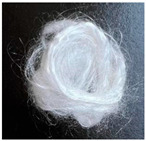	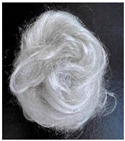	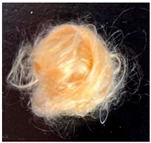	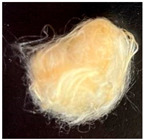

**Table 2:** Summary of photographs of NMMO solutions with *ex situ* synthesized AgNPs used in the fiber production process. The abbreviations correspond to the following: S—NMMO solution. Numbers 12/70 represent synthesis time (h) and temperature (°C), respectively.

Samples	S 0	S-12/70	S-24/70
Color comparison of solutions	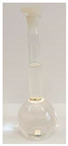	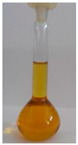	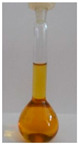

**Table 3:** DLS results for NMMO solutions with *ex situ* synthesized AgNPs and the reference solution (50% NMMO) without AgNPs. The abbreviations correspond to the following: S—NMMO solution. Numbers 12/70 represent synthesis time (h) and temperature (°C), respectively.

Sample	Fraction No.	Volume Weighting		Number Weighting		Intensity Weighting	
		Diameter nm	Percentage %	Diameter nm	Percentage %	Diameter nm	Percentage %
S 0	1	423.7	100	410.5	100	408.4	100
S-12/70	1	11.6	95.3	11.6	99.7	11.1	1.9
	2	119.7	2.4	113.8	0.2	124.5	44.9
	3	912.8	2.4	906.2	<0.1	906.2	53.2
S-24/70	1	12.6	83.1	12.5	97.4	104.9	100
	2	91.4	16.9	88.4	2.6	----	----
	3	----	----	----	----	----	----

**Table 4:** DLS results for cellulose fibers with AgNPs and standard fibers without AgNPs. The abbreviations correspond to the following: F—cellulose fiber; Ag—silver nanoparticles; G—glucose. Numbers 12/70 represent synthesis time (h) and temperature (°C), respectively.

Sample	Fraction No.	Volume Weighting		Number Weighting		Intensity Weighting	
		Diameter nm	Percentage %	Diameter nm	Percentage %	Diameter nm	Percentage %
F 0	1	3.8	100	3.8	100	4.3	100
F Ag/1	1	22.1	98.9	21.9	99.99	23	47.9
	2	1763.5	1.1	1735.4	0.01	1735.5	52.1
	3	---	---	---	---	---	---
F Ag 12/70/G	1	11.8	98.7	11.8	99.9	10.8	15.8
	2	318.7	<0.1	288.9	<0.1	312.8	17.5
	3	3054	1.3	1950.9	<0.1	2950.9	66.8
F Ag 24/70/G	1	12.3	99.8	12.2	99.9	10.9	62.4
	2	1595.6	0.2	1543.8	<0.1	1543.8	37.6
	3	---	---	---	---	---	---

**Table 5:** $\bar{DP}$ for unmodified and AgNP-modified cellulose fibers. The abbreviations correspond to the following: $\bar{DP}$—average degree of polymerization; *SD*—standard deviation; F—cellulose fiber; Ag—silver nanoparticles; G—glucose. Numbers (e.g., 12/70) represent synthesis time (h) and temperature (°C), respectively.

Sample	Fiber Sample Weight, g	$\bar{DP}\pm SD$
F 0	0.0212	1019.18 ± 0.99
F Ag/1	0.0201	1002.70 ± 0.62
F Ag 12/70/G	0.0208	1004.42 ± 0.74
F Ag 24/70/G	0.0202	1004.47 ± 0.91

**Table 6:** Influence of the method of AgNPs generation on the conditioned tenacity and elongation at break of the fibers. The abbreviations correspond to the following: SD—standard deviation; F—cellulose fiber; Ag—silver nanoparticles; G—glucose. Numbers (e.g., 12/70) represent synthesis time (h) and temperature (°C), respectively.

Sample	Linear Density, Dtex	Conditioned Tenacity, cN/tex ± SD	Elongation at Break, % ± SD
F 0	3.01	28.97 ± 6.83	7.79 ± 1.42
F Ag/1	3.04	25.12 ± 7.23	6.24 ± 0.98
F Ag 12/70/G	3.16	23.97 ± 7.73	6.79 ± 1.69
F Ag 24/70/G	3.27	22.66 ± 4.38	6.99 ±1.05

## Data Availability

The original contributions presented in this study are included in the article. Further inquiries can be directed to the corresponding author.
